# Presence of Cu-Type (NirK) and *cd*_1_-Type (NirS) Nitrite Reductase Genes in the Denitrifying Bacterium *Bradyrhizobium nitroreducens* sp. nov.

**DOI:** 10.1264/jsme2.ME18039

**Published:** 2018-09-29

**Authors:** Jeonghwan Jang, Naoaki Ashida, Ayaaki Kai, Kazuo Isobe, Tomoyasu Nishizawa, Shigeto Otsuka, Akira Yokota, Keishi Senoo, Satoshi Ishii

**Affiliations:** 1 Biotechnology Institute, University of Minnesota 140 Gortner Laboratory of Biochemistry, 1479 Gortner Avenue, St. Paul, MN 55108 USA; 2 Department of Applied Biological Chemistry, The University of Tokyo 1–1–1 Yayoi, Bunkyo-ku, Tokyo 113–8657 Japan; 3 Department of Food and Life Sciences, Ibaraki University College of Agriculture 3–21–1 Chuo, Ami, Ibaraki 300–0393 Japan; 4 Collaborative Research Institute for Innovative Microbiology, The University of Tokyo 1–1–1 Yayoi, Bunkyo-ku, Tokyo 113-8657 Japan; 5 Division of Bioscience and Biotechnology for Future Bioindustries, Graduate School of Agricultural Sciences, Tohoku University 468–1 Aramaki Aza Aoba, Aoba-ku, Sendai, Miyagi, 980–0845 Japan; 6 Department of Soil, Water, and Climate, University of Minnesota 439 Borlaug Hall, 1991 Upper Buford Circle, St. Paul, MN 55108 USA

**Keywords:** denitrification, genome, *Bradyrhizobium*, new species

## Abstract

Nitrite reductase is a key enzyme for denitrification. There are two types of nitrite reductases: copper-containing NirK and cytochrome *cd*_1_-containing NirS. Most denitrifiers possess either *nirK* or *nirS*, although a few strains been reported to possess both genes. We herein report the presence of *nirK* and *nirS* in the soil-denitrifying bacterium *Bradyrhizobium* sp. strain TSA1^T^. Both *nirK* and *nirS* were identified and actively transcribed under denitrification conditions. Based on physiological, chemotaxonomic, and genomic properties, strain TSA1^T^ (=JCM 18858^T^=KCTC 62391^T^) represents a novel species within the genus *Bradyrhizobium*, for which we propose the name *Bradyrhizobium nitroreducens* sp. nov.

Denitrification is a microbial respiratory process in which nitrogen oxides, such as nitrate and nitrite, are reduced in a stepwise manner to gaseous nitrogen products (NO, N_2_O, and N_2_) (36). Each step in denitrification is catalyzed by distinct enzymes: nitrate reductase (Nar), nitrite reductase (Nir), nitric oxide reductase (Nor), and nitrous oxide reductase (Nos). There are two types of nitrite reductases: the copper-containing NirK type and cytochrome *cd*_1_-containing NirS type (36). The genes encoding these enzymes (*nirK* and *nirS*) have been frequently used as markers to identify denitrifiers (2). Although a few strains have been reported to harbor both *nirK* and *nirS* (3, 6, 22), most denitrifiers possess either *nirK* or *nirS*.

We previously isolated several *Bradyrhizobium* sp. strains that possess *nirS* from rice paddy soil in Japan (8). We then isolated additional *nirS*-harboring *Bradyrhizobium* sp. denitrifiers from other soils (15). The presence of *nirS* in *Bradyrhizobium* spp. is unique because most of the other *Bradyrhizobium* strains possess *nirK* (1, 21, 22). In addition, these *nirS* sequences form distinct clusters from other *nirS* sequences (8). Most of the *nirK* and *nirS* sequences (*e.g.*, 16–42% and 15–35%, respectively) obtained by culture-independent analyses were found to be closely related to the *nirK* and *nirS* of the *Bradyrhizobium* sp. denitrifier (Fig. S1) (32, 33), suggesting an important role for *Bradyrhizobium* spp. in denitrification in the environment. Based on our preliminary PCR examination, we detected *nirK* in some *nirS*-positive *Bradyrhizobium* strains. In order to further clarify whether these strains possess both *nirK* and *nirS*, these genes need to be identified by whole genome sequencing.

We herein report the presence of both *nirK* and *nirS* in *Bradyrhizobium* sp. denitrifiers based on a genome analysis and transcription experiments. We further analyzed the physiological traits, such as nitrogen fixation and nodulation abilities, of the representative strain TSA1^T^. The results of these analyses indicated that *nirK-* and *nirS*-positive *Bradyrhizobium* strains were taxonomically distinct from any of the previously established species of the genus *Bradyrhizobium*. Thus, we propose *Bradyrhizobium nitroreducens* sp. nov. for strain TSA1^T^. Strain TSA1^T^ was selected as the representative of *nirK-* and *nirS*-positive *Bradyrhizobium* based on a phylogenetic analysis targeting the 16S rRNA gene and internal transcribed spacer (ITS) sequences (8).

Strain TSA1^T^ was originally isolated from rice paddy soil collected at the Institute for Sustainable Agro-Ecosystems, the Graduate School of Agricultural and Life Sciences, The University of Tokyo (Nishitokyo, Tokyo, Japan; 35°44′ N, 139°32′ E) (8). The culture of strain TSA1^T^ was maintained on 100-fold diluted nutrient broth (DNB) (BD Difco™; Becton, Dickinson and Company, Franklin, Lakes, NJ, USA) supplemented with 3 mM nitrate and 4.4 mM succinate (DNBNS medium) or 1.5% agar plates of DNBNS medium (DNBNS agar medium) under an anoxic incubation at 30°C as described previously (8). Flagellar motility was assayed on semi-solid DNBNS medium containing 0.075% agar (11). In fatty acid and quinone analyses, cells were aerobically grown on R2A agar (BD Difco™) and R2A liquid medium, respectively, and harvested at the exponential phase of growth. Cellular fatty acids were analyzed by gas chromatography, as previously described (11). Quinones were analyzed by reverse-phase HPLC as described elsewhere (13). Growth at different temperatures, pH, and antibiotic supplement conditions was examined in R2A liquid medium under an aerobic incubation at 30°C. In the carbon utilization test, modified yeast extract (YE) medium containing a minimal amount of yeast extract (10 mg L^−1^) and 10 g L^−1^ carbon source was used as described previously (28). The growth rates of the strain were measured in R2A broth medium and yeast extract mannitol (YEM) broth medium (27) at 30 and 37°C. Cell morphology was observed using the Philips CM12 transmission electron microscope (TEM) after cells were negatively stained with 1% of aqueous potassium phosphotungstic acid (pH 7.0).

Potential nitrogen fixation ability was examined by measuring acetylene reduction activity as described previously (11). Denitrification ability was analyzed using the acetylene block method as described previously (23). N_2_O-reducing ability was examined using ^15^N-labeled N_2_O (^15^N, 99 atom. %; Cambridge Isotope Laboratories) and gas chromatography/mass spectrometry (GC/MS) as described previously (9). A nodulation assay was performed using siratro as the host plant as described elsewhere (20). *Bradyrhizobium diazoefficiens* USDA110^T^ was used as the positive control for these assays.

The genomic DNA of strain TSA1^T^ was extracted for genome sequencing as previously described (14). The library was prepared using the TruSeq DNA sample prep kit (Illumina, San Diego, CA, USA) and sequenced using the Illumina HiSeq 2000 platform with 101-bp paired-end sequencing chemistry. Sequence reads were assembled using Velvet v. 12.0.8 (35) followed by gene annotation performed with the NCBI Prokaryotic Genome Annotation Pipeline (25). A genome sequence and annotation summary is shown in Table S1. Average nucleotide identity (ANI) values were calculated using JSpecies (19) in order to examine the relatedness of strain TSA1^T^ and its close relatives. We also calculated the percentage of conserved proteins (POCP) as described by Qin *et al.* (17).

A phylogenetic analysis was performed using the 16S rRNA gene, ITS, *nirK*, and *nirS* sequences in order to examine sequence relatedness among *Bradyrhizobium* and other denitrifying strains. Multilocus sequencing typing (MLST) was also performed using concatenated sequences of *glnII*, *recA*, *rpoB*, and *dnaK* (7). Phylogenetic trees were generated using the neighbor-joining and maximum-likelihood methods with a bootstrap analysis (*n*=1,000) using MEGA6 software (24).

In order to examine whether *nirK* and *nirS* are transcribed under denitrification conditions, we performed reverse transcription quantitative PCR (RT-qPCR) targeting these gene transcripts as well as 16S rRNA. RNA was isolated from TSA1^T^ cells grown in R2A broth supplemented with 10 mM acetate and 5 mM nitrate under oxic and anoxic conditions for 3, 6, 12, and 24 h. RNA was also isolated from preculture cells grown in the same medium but without nitrate under oxic conditions (=0-h samples). The absence of genomic DNA in RNA samples was verified by PCR targeting the 16S rRNA gene as described previously (7). The primers nirSCd3aF and nirSR3cd were used for the quantification of *nirS* (12), while we designed the new primers BRnirK_F (5′-TTCGT CTATCACTGCGCC-3′) and BRnirK_R (5′-CAGCTTCTT CATCACCTCTTC-3′) for the quantification of *nirK* because currently available primers had several base mismatches to the *nirK* of strain TSA1^T^. RNA was reverse transcribed using the PrimeScript RT reagent kit (Takara Bio, Otsu, Shiga, Japan) with random hexamers as described previously (10). The reaction mixture for RT-qPCR contained 1× SYBR Premix ExTaq ROXplus (Takara Bio), 0.2 μM of each primer, and 100 ng cDNA. RT-qPCR was performed using the StepOnePlus Real-Time PCR System (v. 2.3; Applied Biosystems, Foster City, CA, USA) under the following conditions: 95°C for 30 s, followed by 40 cycles of 95°C for 5 s and 60°C for 30 s. Transcription levels were normalized using the quantity of 16S rRNA.

The morphological, cultural, physiological, and biochemical characteristics of the *nirK* and *nirS*-harboring *Bradyrhizobium* strains represented by strain TSA1^T^ are summarized in Table 1. Cells were rod-shaped, measuring 0.4–0.6 μm in width and 1.6–2.2 μm in length, and were motile with a single polar flagellum (Fig. S4). The colonies that formed on R2A agar medium were smooth, circular, white, and convex. Cells of strain TSA1^T^ grew in a temperature range of 25–37°C (optimum, 30°C) in R2A and YEM media. The doubling times of TSA1^T^ in R2A and YEM media at 30°C were 10.6 and 11.2 h, respectively, similar to other *Bradyrhizobium* strains (34). Cells also grew under a wide range of pH conditions (pH 4.5–10.0) similar to *B. ottawaense* O99^T^, but dissimilar to the other *Bradyrhizobium* strains. Unlike *B. ottawaense* O99^T^, strain TSA1^T^ did not grow in media containing >1% NaCl (w/v). Strain TSA1^T^ showed resistance to 100 μg mL^−1^ ampicillin, 20 μg mL^−1^ tetracycline, and 300 μg mL^−1^ polymyxin. Resistance to these antibiotics made TSA1^T^ distinct from other closely related *Bradyrhizobium* strains. Growth was inhibited by 100 μg mL^−1^ erythromycin and 50 μg mL^−1^ kanamycin. Cells grew using d- and l-arabinose, d-fructose, d-glucose, maltose, d-mannitol, d-mannose, l-rhamnose, gluconate, d-sorbitol, and d-xylose. Growth was not observed in YE medium supplemented with cellobiose, inositol, d-lactose, and d-sucrose.

Strain TSA1^T^ exhibited the ability to grow under anoxic conditions with nitrate as the terminal electron acceptor as well as under aerobic conditions. They also reduced exogenous N_2_O to N_2_. The presence of all of the functional genes for denitrification, including *napA*, *nirK*, *nirS*, *norB*, and *nosZ*, on an 8.1-Mbp contig of the genome (Contig01) of strain TSA1^T^ (Table S2) supported this result.

Based on RT-qPCR results, *nirK* and *nirS* of strain TSA1^T^ were both actively transcribed under anoxic conditions in the presence of nitrate (*i.e.*, denitrification conditions), although the number of *nirK* transcripts was always higher than that of *nirS* transcripts (Fig. 1). The transcription of *nirK* and *nirS* was the most intensive under denitrification conditions at the first time point (3 h), and decreased thereafter. The transcription of *nirK* and *nirS* was suppressed when oxygen was present and nitrate was absent. Although the addition of nitrate increased the transcription levels of *nirK* and *nirS* under oxic conditions, transcription levels were still significantly lower than those under anoxic conditions (*P*<0.05 by ANOVA). These results suggest that *nirK* and *nirS* in strain TSA1^T^ are regulated in response to the availability of oxygen and nitrate. The occurrence of both *nirK* and *nirS* in *B. oligotrophicum* S58^T^ (formally known as *Agromonas oligotropha*) (16) was recently reported (22). The findings reported by Sanchez and Minamisawa (22) suggest that NirK-type and NirS-type nitrite reductases are functionally redundant in strain S58^T^; however, the functional redundancy of nitrite reductases in strain TSA1^T^ remains unknown.

Genes related to nitrogen fixation, such as *fixR*, *nifA*, *sufBCDSE*, *nifDKENX*, *nifHV*, and *fixABC*, were identified on the genome of strain TSA1^T^. These gene clusters are highly conserved among symbiotic nitrogen-fixing bradyrhizobia (16). The nitrogen-fixing ability of strain TSA1^T^ was confirmed in an acetylene reduction assay. Strain TSA1^T^ lacks the common nodulation gene cluster *nodABC* on its genome, and did not nodulate siratro (data not shown). In addition, the photosynthetic gene cluster (*pufBALM*) was not detected on the genome of strain TSA1^T^. These results suggest that strain TSA1^T^ is a nitrogen-fixing, non-phototrophic, free-living *Bradyrhizobium*.

Strain TSA1^T^ was the most closely related to *B. diazoefficiens* USDA110^T^ based on the 16S rRNA gene sequence analysis, with a similarity value of 99.7% (Fig. S2); however, strain TSA1^T^ was more closely related to *B. liaoningense* CCNWSX0360 and *B. yuanmingense* CCBAU10071^T^ based on an analysis of ITS sequences (Fig. 2A). The MLST analysis based on four housekeeping genes suggested that strain TSA1^T^ was also closely related to *B. ottawaense* O99^T^ (Fig. S3). The *nirK* sequence of strain TSA1^T^ was also closely related to those of *B. ottawaense*, *B. diazoefficiens*, *B. liaoningense*, and *B. yuanmingense* (Fig. 2B). While *B. oligotrophicum* S58^T^ also possesses the *nirK* and *nirS* homologs, this strain was relatively distantly related to strain TSA1^T^ based on the 16S rRNA gene, ITS, and *nirK* sequences. In contrast, *nirS* sequences from strain TSA1^T^ and *B. oligotrophicum* S58^T^ were similar to each other with 87% sequence similarity (Fig. 2C). These phylogenetic analyses suggest that strain TSA1^T^ has a common evolutionary history with *B. diazoefficiens*, *B. liaoningense*, *B. yuanmingense*, and *B. ottawaense*, and *nirS* was horizontally acquired from elsewhere by strain TSA1^T^ and *B. oligotrophicum* S58^T^. We attempted to provide evidence of horizontal gene transfer (*e.g.*, integrons, phages, insertion sequences, and genomic islands) around *nirS*, but were unsuccessful.

The average nucleotide identity (ANI) values between strain TSA1^T^ and closely related *Bradyrhizobium* species (selected based on the 16S rRNA and ITS phylogenies) ranged between 61 and 86% (Table S3). These ANI values were smaller than 95%, which is recommended as the species boundary (5), indicating that strain TSA1^T^ needs to be classified as a novel species within the genus *Bradyrhizobium*. The POCP value between strain TSA1^T^ and most of the other closely related *Bradyrhizobium* species ranged between 54 and 69% (Table S4). The POCP value of 50% was proposed as the cut-off for the genus (17), supporting the affiliation of strain TSA1^T^ to the genus *Bradyrhizobium*. The POCP value between strain TSA1^T^ and *B. oligotrophicum* S58^T^ was smaller than the genus cut-off value of 50% (37.08%, Table S4). However, *B. oligotrophicum* S58^T^ also had low POCP values with other *Bradyrhizobium* species, suggesting that *B. oligotrophicum* S58^T^ had different gene contents from other *Bradyrhizobium* species, which is consistent with the phylogenetic analysis based on the four housekeeping genes (Fig. S3).

The G+C content of strain TSA1^T^ was 64.3% based on draft genome sequencing. This value is similar to those of other closely related *Bradyrhizobium* spp. (Table 1). Strain TSA1^T^ had Q-10 as the only ubiquinone, similar to other *Bradyrhizobium* spp. (29). The fatty acid profile of TSA1^T^ contained 75.8% C_18:1_ ω7c, 15.0% C_16:0_, 5.9% C_18:0_, 2.0% C_12:0_, and 1.3% C_14:0_, which is similar to other *Bradyrhizobium* spp. (26). These results support the affiliation of strain TSA1^T^ to the genus *Bradyrhizobium*.

In conclusion, the group of *Bradyrhizobium* sp. strains represented by strain TSA1^T^ is unique, in that they possess both Cu-type and cytochrome *cd*_1_-type nitrite reductase genes (*nirK*- and *nirS*). Although *nirK* and *nirS* were also found on the genome of *B. oligotrophicum* S58^T^, strain TSA1^T^ is phylogenetically distantly related to strain S58^T^. Since a large proportion of *nirK* and *nirS* clone libraries was related to the nitrite reductase genes of *Bradyrhizobium* sp. TSA1^T^ (32, 33), these bacteria may play an important role in denitrification in soil. Based on the 16S rRNA gene sequence analysis in combination with physiological, chemotaxonomic, and genomic properties, strain TSA1^T^ (=JCM 18858^T^=KCTC 62391^T^) represents a novel species within the genus *Bradyrhizobium*, for which we propose the name *Bradyrhizobium nitroreducens* sp. nov.

## Description of *Bradyrhizobium nitroreducens* sp. nov.

*Bradyrhizobium nitroreducens* (ni.tro.re.du’cens. Gr. n. *nitron* nitrate, nitrite; L. adj. from pres. part. of verb *reduco* reduce, bring back to a condition; N.L. adj. *nitroreducens* reducing nitrate and nitrite).

Cells are Gram-negative rods measuring 0.4–0.6 μm in width and 1.6–2.2 μm in length. Motile by means of a polar flagellum. Colonies formed on R2A agar medium are smooth, circular, white, and convex. Facultative anaerobic. Cells grow with oxygen, nitrate, or N_2_O as the terminal electron acceptor. Denitrification positive. Cells possess both *nirK* and *nirS* as the nitrite reductase gene. Nitrogen fixation positive. Cells do not form nodules to siratro. Cells have the ability to grow at 25–37°C (optimum 30°C) and pH 4.5–10. No growth occurs in the presence of 1% NaCl in R2A medium. Cells are resistant to ampicillin (100 μg mL^−1^), tetracycline (20 μg mL^−1^), and polymyxin (300 μg mL^−1^), but sensitive to erythromycin (100 μg mL^−1^) and kanamycin (50 μg mL^−1^). Usable carbon and energy sources are d- and l-arabinose, d-fructose, d-glucose, maltose, d-mannitol, d-mannose, l-rhamnose, gluconate, d-sorbitol, and d-xylose. Cellobiose, inositol, d-lactose, and d-sucrose are not used. The major cellular fatty acids (>10% of total fatty acids) are C_16:0_ and C_18:1_ω7c. The predominant quinone is ubiquinone-10. The type strain TSA1^T^ (=JCM 18858^T^=KCTC 62391^T^) was isolated from rice paddy soil in Tokyo, Japan. The DNA G+C content of the type strain is 64.3% as calculated from draft genome sequencing.

The GenBank/EMBL/DDBJ accession numbers for the 16S rRNA gene sequence and the genome sequence of strain TSA1^T^ are AB542368 and LFJC00000000, respectively.

## Supplementary Information



## Figures and Tables

**Fig. 1:**
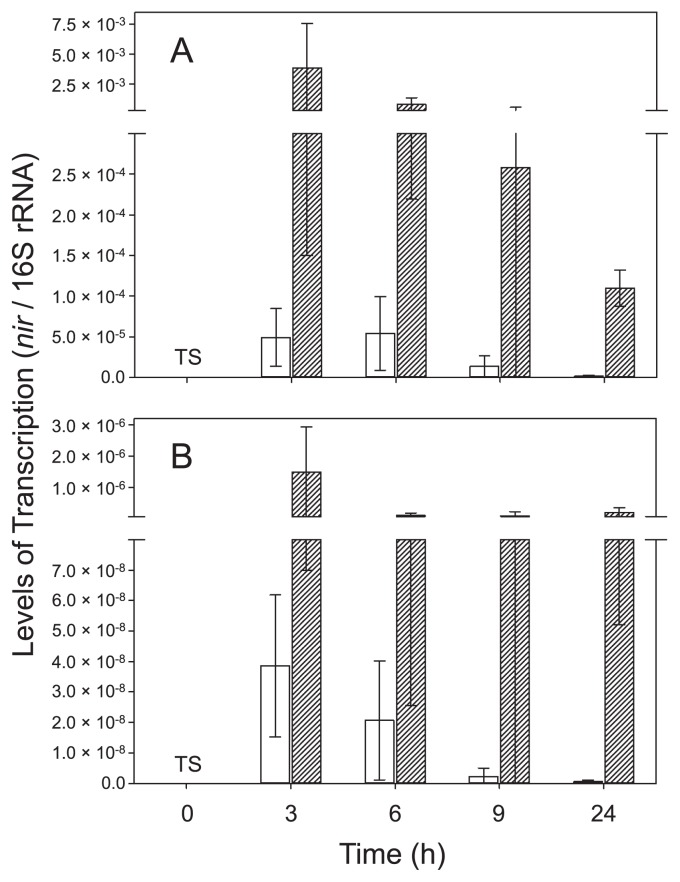
Transcription levels of (A) *nirK* and (B) *nirS* in *Bradyrhizobium nitroreducens* strain TSA1^T^ under oxic and anoxic conditions. Transcription levels were normalized by the amount of 16S rRNA. Legend: □, oxic; ▨, anoxic; TS, too small to show. Levels of transcription at 0 h (=preculture) were 1.46×10^−8^ and 1.47×10^−11^ for *nirK* and *nirS*, respectively.

**Fig. 2:**
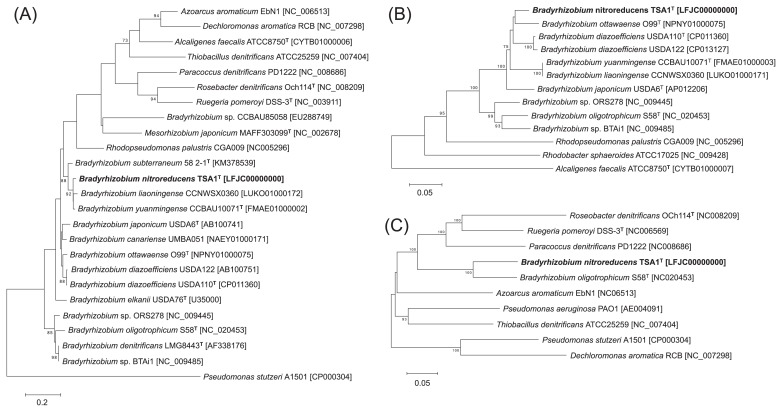
Phylogenetic relationship of denitrifying strains based on (A) ITS, (B) *nirK*, and (C) *nirS* sequences. Trees were constructed by the neighbor-joining method using MEGA6 software (17). GenBank accession numbers are shown in square brackets. Bootstrap values (%) were generated from 1,000 replicates, and values >70% are shown. Branch lengths correspond to sequence differences as indicated by the scale bar.

**Table 1:** Phenotypic, genetic, and chemotaxonomic characteristics that separate strain TSA1^T^ from closely related phylogenetic neighbors. Strains: 1, TSA1^T^; 2, *B. yuanmingense* CCBAU 10071^T^; 3, *B. liaoningense* 2281^T^; 4, *B. diazoefficiens* USDA110^T^; 5, *B. ottawaense* O99^T^; 6, *B. oligotropha* S58^T^.

Characteristic	1	2	3	4	5	6
Denitrification	+	ND	ND	+	−	+
*nir* type	*nirK*, *nirS*	*nirK*	*nirK*^a^	*nirK*	*nirK*	*nirK*, *nirS*
Nitrogen fixation	+	+	+	+	+	+
Nodulation	−	+	+	+	+	+
Major FA	16:0, 18:1 ω7c	ND	ND	16:0, 18:1 ω6c/ω7c	16:0, 18:1 ω6c/ω7c	18:1
Major quinone	Q10	ND	ND	Q10^b^	ND	Q10
DNA G+C content (mol %)	64.3	62–64	60–64	64	62.6	65.1
Carbon source utilization						
d-Arabinose	+	W	W	+	ND	ND
l-Arabinose	+	+	+	W	+	+
Cellobiose	−	−	−	−	ND	−
d-Fructose	+	W	−	W	ND	ND
d-Glucose	+	W	−	W	+	+
Inositol	−	−	−	−	ND	−
d-Lactose	−	−	−	+	−	−
Maltose	+	W	−	−	+	−
d-Mannitol	+	W	W	W	+	+
d-Mannose	+	W	+	W	+	+
l-Rhamnose	+	W	W	W	+	+
Sodium gluconate	+	−	W	−	+	+
d-Sorbitol	W	−	−	W	ND	−
d-Sucrose	−	−	−	W	ND	−
d-Xylose	+	+	+	W	+	+
Growth in/at						
37°C	+	+	−	−	+	+
pH 4.5	+	W	W	+	+	ND
pH 8.0	+	+	+	−	+	ND
pH 10.0	+	−	−	−	+	ND
1% NaCl	−	−	−	−	+	−
Resistance (μg mL^−1^)						
Polymyxin (300)	+	+	ND	−	ND	ND
Erythromycin (100)	−	−	−	−	+	ND
Tetracycline (20)	+	+	W	−	+	ND
Ampicillin (10)	+	−	−	W	−	ND

Reference	This study	(4, 31)	(4, 30)	(4)	(34)	(16, 18)

a Based on the genome of strain CCNWSX0360 (GenBank accession LUKO01000171)

b Assessed in this study.

+, growth; −, no growth; w, weakly positive; ND, not determined.
