# Supplementary material for: Presence of Cu-Type (NirK) and cd1-Type (NirS) Nitrite Reductase Genes in the Denitrifying Bacterium Bradyrhizobium nitroreducens sp. nov
Source: Microbes Environ. 2018 Sep 29;33(3):326–31. doi: 10.1264/jsme2.ME18039 (PMC6167111; doi:10.1264/jsme2.ME18039)

Supplementary Information

# Presence of both Cu-type (NirK) and $cd_1$-type (NirS) nitrite reductase genes in soil denitrifying bacterium *Bradyrhizobium nitroreducens* sp. nov.

JEONGHWAN JANG, NAOAKI ASHIDA, AYAAKI KAI, KAZUO ISOBE, TOMOYASU NISHIZAWA, SHIGETO OTSUKA, AKIRA YOKOTA, KEISHI SENOO, SATOSHI ISHII*

*Corresponding author. Email: ishi0040@umn.edu

Table S1. Genome sequencing summary for *Bradyrhizobium nitroreducens* strain TSA1$^T$.

| Attribute | Value |
|---|---|
| Total sequence reads | 1,729 Mbp |
| Number of contigs | 161 |
| Total length of contigs | 8,213,033 bp |
| Max length of contig | 8,173,757 bp |
| Genome coverage | 210× |
| Number of CDSs | 7,268 |
| Number of tRNAs | 50 |
| Number of rRNA genes (5S, 16S, 23S) | 3 (1, 1, 1) |
| GenBank accession No. | LFJC00000000 |

Table S2. Functional genes for denitrification and nitrogen fixation found in the genome of *Bradyrhizobium nitroreducens* TSA1$^{T}$.

| Pathway | locus_tag | Gene | Related product |
|---|---|---|---|
| Denitrification | ACG08_10910 | *napB* | Nitrate reductase |
| | ACG08_10915 | *napH* | |
| | ACG08_10920 | *napG* | |
| | ACG08_10925 | *napA* | |
| | ACG08_11155 | *nirK* | Nitrite reductase |
| | ACG08_11160 | *nirV* | |
| | ACG08_18500 | *nirN* | Nitrite reductase |
| | ACG08_18505 | *nirJ* | |
| | ACG08_18510 | *nirH* | |
| | ACG08_18515 | *nirG* | |
| | ACG08_18520 | *nirD* | |
| | ACG08_18525 | *nirF* | |
| | ACG08_18530 | *nirC* | |
| | ACG08_18535 | *nirE* | |
| | ACG08_18540 | *nirS* | |
| | ACG08_18545 | *nirI* | |
| | ACG08_18550 | *nirX* | |
| | ACG08_19020 | *norD* | Nitric oxide reductase |
| | ACG08_19025 | *norQ* | |
| | ACG08_19030 | *norB* | |
| | ACG08_19035 | *norC* | |
| | ACG08_19040 | *norF* | |
| | ACG08_19045 | *norE* | |
| | ACG08_16845 | *nosR* | Nitrous oxide reductase |
| | ACG08_16850 | *nosZ* | |
| | ACG08_16855 | *nosD* | |
| | ACG08_16860 | *nosF* | |
| | ACG08_16865 | *nosY* | |
| | ACG08_16870 | *nosL* | |
| | ACG08_16875 | *nosX* | |
| Nitrogen fixation | ACG08_08410 | *nifA* | ATPase AAA oxidoreductase |
| | ACG08_08415 | *fixR* | |
| | ACG08_24230 | *sufB* | Cysteine desulfurase |
| | ACG08_24235 | *sufC* | |
| | ACG08_24240 | *sufD* | |
| | ACG08_24245 | *sufS* | |
| | ACG08_24255 | *sufE* | |
| | ACG08_24260 | *nifD* | Nitrogenase reductase |
| | ACG08_24265 | *nifK* | |
| | ACG08_24270 | *nifE* | |
| | ACG08_24275 | *nifN* | |
| | ACG08_24280 | *nifX* | |
| | ACG08_24465 | *nifH* | |
| | ACG08_24480 | *nifV* | |
| | ACG08_24495 | *fixA* | |
| | ACG08_24500 | *fixB* | |
| | ACG08_24505 | *fixC* | |

Table S3. Average nucleotide identity (ANI) values between *Bradyrhizobium nitroreducens* strain TSA1$^T$ and other *Bradyrhizobium* spp.

| Compared strains | ANI value (%) |
|---|---|
| *Bradyrhizobium arachidis* LMG26795$^T$ | 64.11 |
| *Bradyrhizobium canariense* UMBA050 | 85.14 |
| *Bradyrhizobium diazoefficiens* USDA110$^T$ | 65.59 |
| *Bradyrhizobium liaoningense* CCNWSX0360 | 73.28 |
| *Bradyrhizobium japonicum* USDA6$^T$ | 65.59 |
| *Bradyrhizobium manausense* BR3351$^T$ | 61.82 |
| *Bradyrhizobium oligotrophicum* S58$^T$ | 77.32 |
| *Bradyrhizobium ottawaense* O99$^T$ | 80.0 |
| *Bradyrhizobium yuanmingense* CCBAU10071$^T$ | 71.37 |

Table S4. Percentage of conserved proteins (POCP) values between *Bradyrhizobium nitroreducens* TSA1$^T$, *Bradyrhizobium oligotrophicum* S58$^T$, and other *Bradyrhizobium* spp.

| Compared strains | *Bradyrhizobium nitroreducens* TSA1$^T$ | *Bradyrhizobium oligotrophicum* S58$^T$ |
|---|---|---|
| *Bradyrhizobium nitroreducens* TSA1$^T$ | - | 37.08 |
| *Bradyrhizobium arachidis* LMG26795$^T$ | 63.86 | 33.60 |
| *Bradyrhizobium canariense* UMBA050 | 60.79 | 31.61 |
| *Bradyrhizobium diazoefficiens* USDA110$^T$ | 66.11 | 34.41 |
| *Bradyrhizobium liaoningense* CCNWSX0360 | 64.48 | 33.29 |
| *Bradyrhizobium japonicum* USDA6$^T$ | 61.44 | 32.09 |
| *Bradyrhizobium manausense* BR3351$^T$ | 54.01 | 30.09 |
| *Bradyrhizobium ottawaense* O99$^T$ | 68.69 | 33.22 |
| *Bradyrhizobium yuanmingense* CCBAU10071$^T$ | 63.58 | 33.29 |
| *Bradyrhizobium oligotrophicum* S58$^T$ | 37.08 | - |

Figure S1. Proportion of (A) *nirK* and (B) *nirS* clones closely related to the *nirK* of *nirS* sequences of *Bradyrhizobium* sp. denitrifiers (black fill) in the clone libraries, respectively. Figure modified from Yoshida et al. (26).

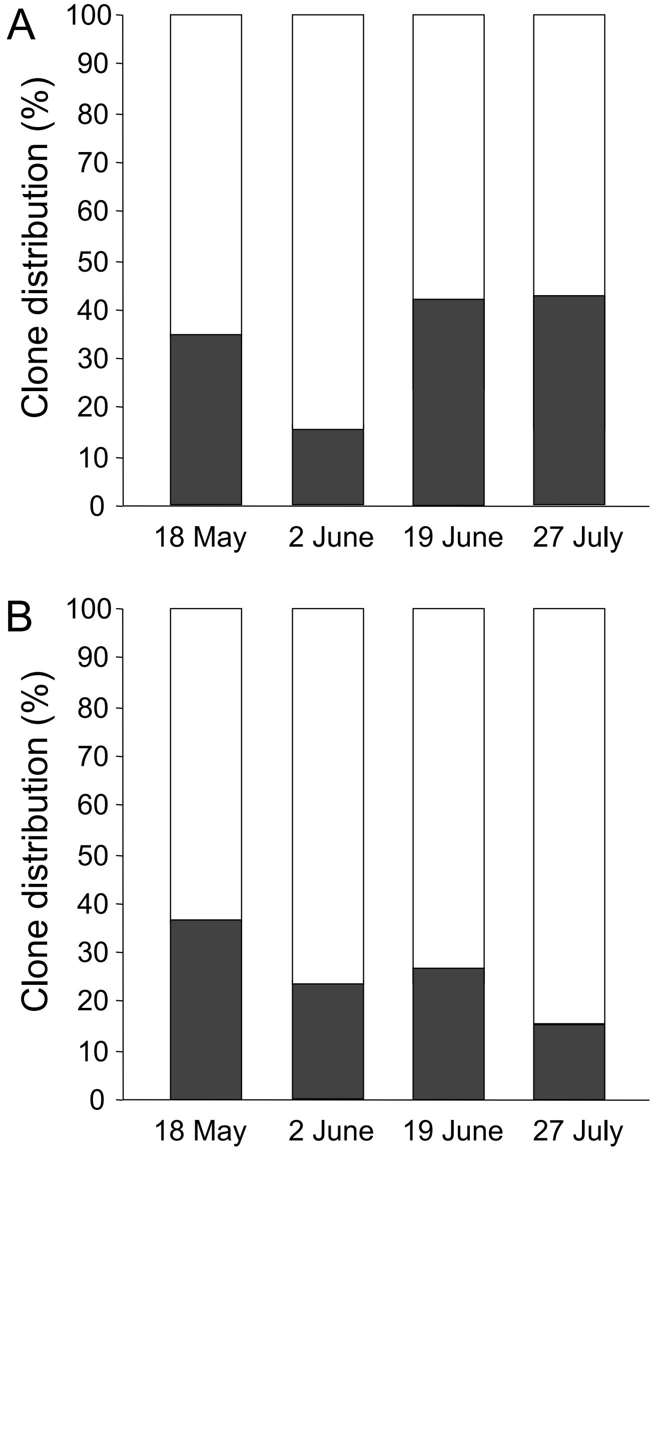

Figure S2. Phylogenetic relationship of *Bradyrhizobium* species based on the 16S rRNA gene sequences. The tree was constructed by the neighbor-joining method using MEGA6 software. GenBank accession numbers are shown in square brackets. Bootstrap values (%) were generated from 1000 replicates, and the values >70% are shown. Branch lengths correspond to sequence differences as indicated by the scale bar.

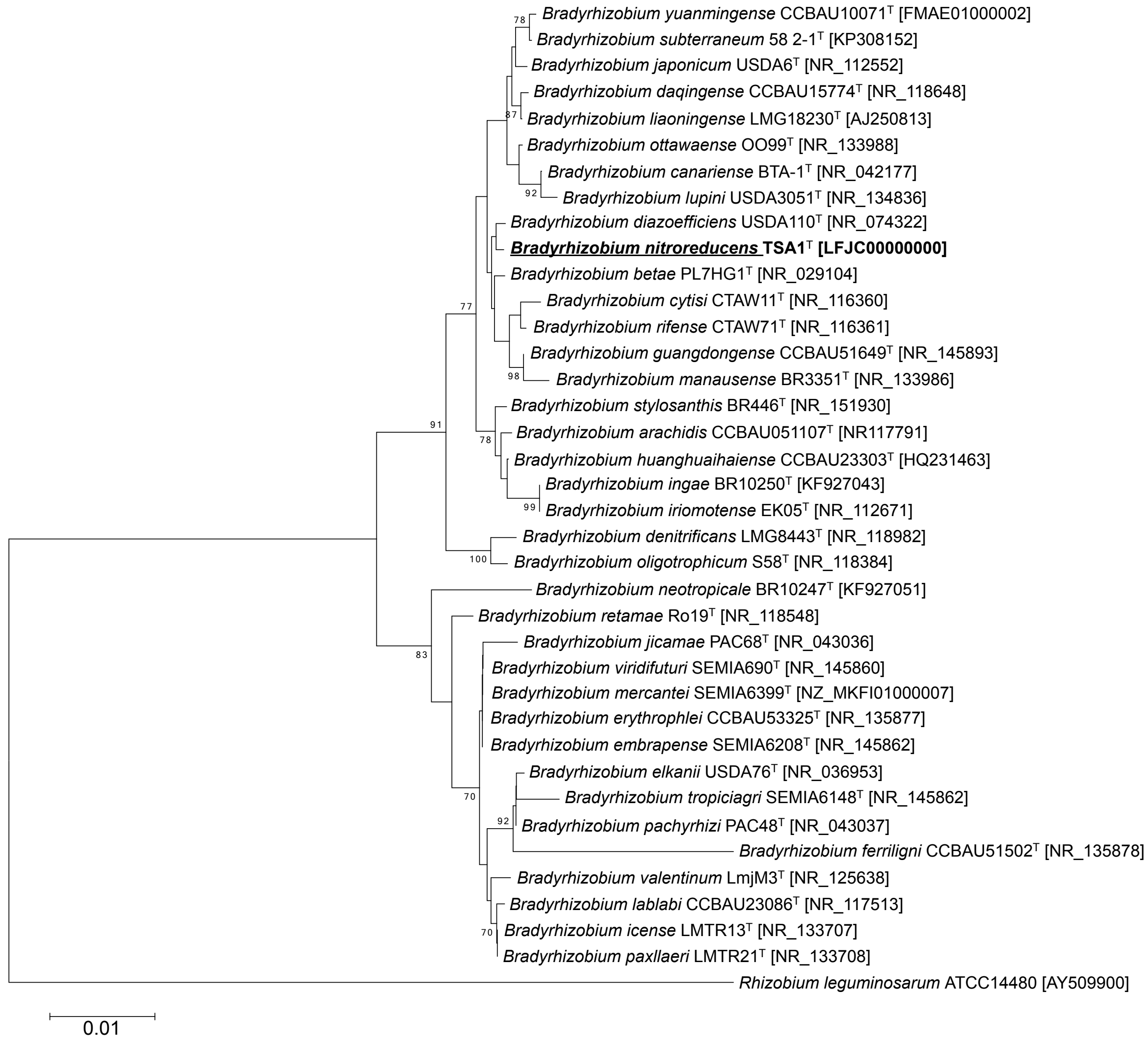

Figure S3. Phylogenetic relationship of *Bradyrhizobium* species based on the sequences of the *glnII*-*recA*-*rpoB*-*dnaK* concatemers. The tree was constructed by using maximum-likelihood method with the GTR + G + I model and MEGA6 software. GenBank accession numbers are shown in square brackets. Bootstrap values (%) were generated from 1000 replicates, and the values >70% are shown. Branch lengths correspond to sequence differences as indicated by the scale bar.

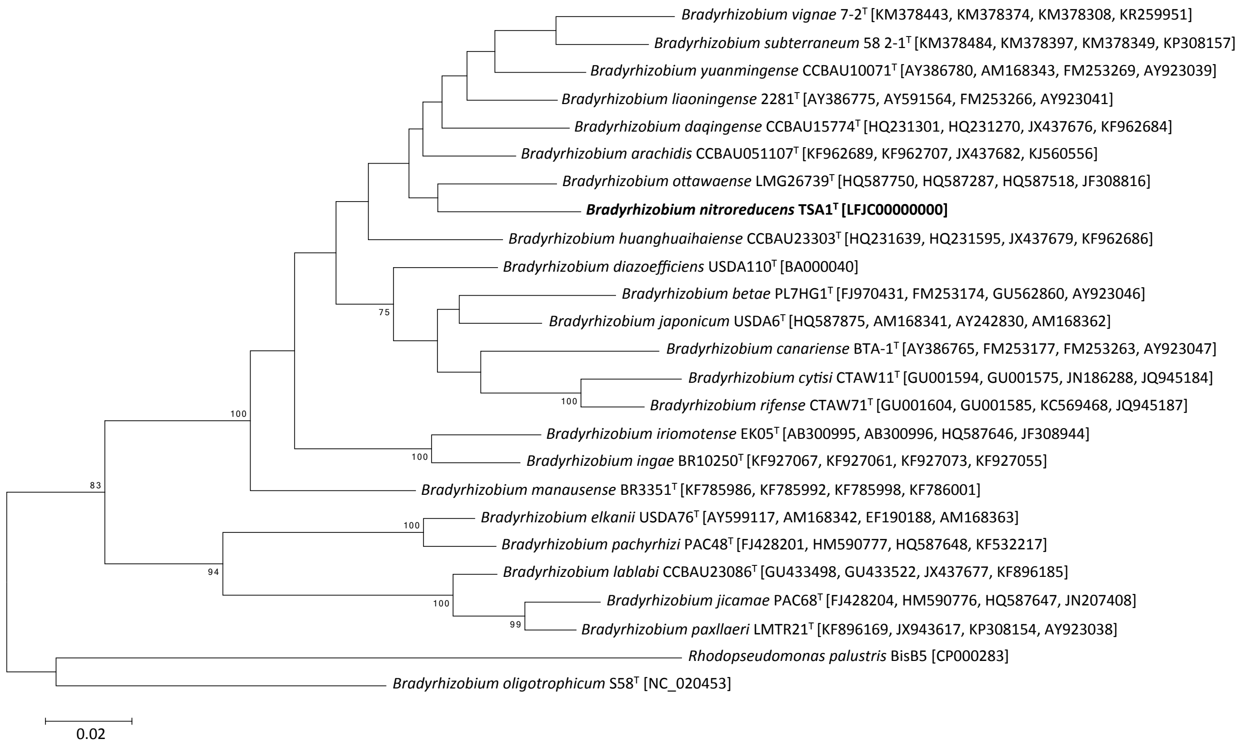

Figure S4. Transmission electron microscopic image of a cell of *Bradyrhizobium nitroreducens* TSA1$^{T}$.

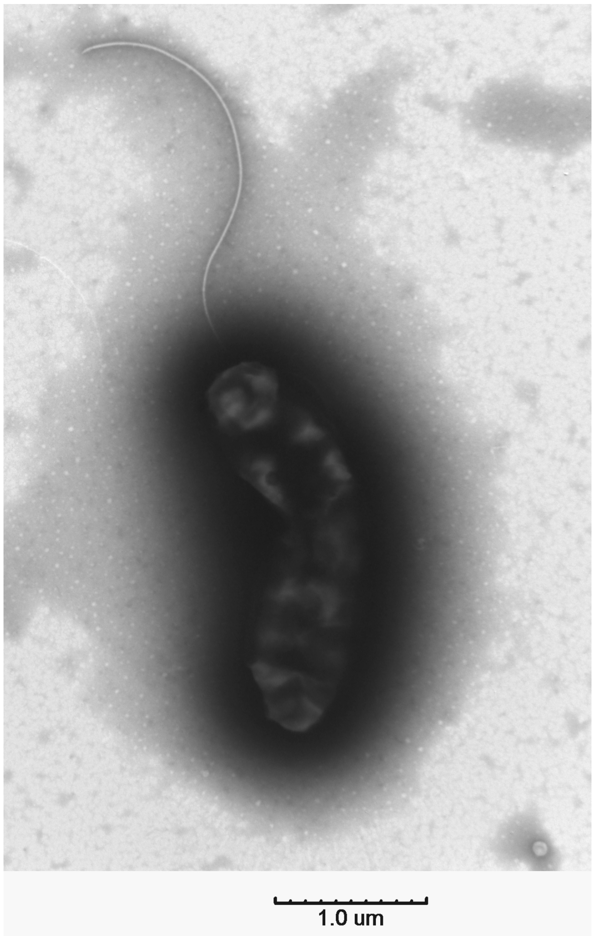

Supplement: Supplementary file 1 [file 33_326_s1.pdf]
